# Characterization of B-cell and T-cell responses to a tetravalent dengue purified inactivated vaccine in healthy adults

**DOI:** 10.1038/s41541-022-00537-2

**Published:** 2022-10-31

**Authors:** Heather Friberg, Morgan Gargulak, Amanda Kong, Leyi Lin, Luis J. Martinez, Alexander C. Schmidt, Robert M. Paris, Richard G. Jarman, Clemente Diaz, Stephen J. Thomas, Philippe Moris, Jeffrey R. Currier

**Affiliations:** 1 Viral Diseases Branch, Walter Reed Army Institute of Research, Silver Spring, MD USA; 2 GSK, Rockville, MD USA; 3 University of Puerto Rico School of Medicine, San Juan, Puerto Rico; 4 GSK, Rixensart, Belgium

**Keywords:** Inactivated vaccines, Viral infection

## Abstract

The increasing global impact of dengue underscores the need for a dengue virus (DENV) vaccine. We assessed B-cell and T-cell responses following vaccination with four formulations of a tetravalent dengue purified inactivated vaccine (DPIV) in dengue-primed and dengue-naive adults from two studies (NCT01666652, NCT01702857). Frequencies of DPIV-induced memory B cells specific to each DENV serotype remained high up to 12 months post-vaccination, and were higher in the dengue-primed than dengue-naive adults. A subsequent DPIV booster dose induced strong anamnestic B-cell responses. Multifunctional CD4+ T cells (predominantly expressing IL-2) were induced by DPIV, with higher frequencies in dengue-primed adults. DPIV-induced CD4+ T cells also demonstrated in vitro proliferative capacity and antigen-specific production of GM-CSF, IFN-γ, and IL-13. CD8+ T-cell responses were undetectable in dengue-naive adults and low in dengue-primed individuals. B- and T-cell responses persisted up to 12 months post-vaccination in both dengue-primed and dengue-naive adults.

## Introduction

Dengue is a viral disease manifesting as an influenza-like illness, and which in severe cases can become life-threatening, with case fatality rates up to 2.5% estimated worldwide^1,2^. The incidence of dengue continues to rise due to a number of factors, including increasing global travel trends and vector spread. The number of reported dengue cases has risen from over 1.2 million in 2008 to 3.34 million in 2016 in the Americas, South-East Asia, and Western Pacific regions only^2^. Moreover, additional outbreaks are reported each year, including in regions previously unaffected, such as Europe^3^. The associated global economic burden of dengue is high^4^, estimated at $8.9 billion for 2013^5^. However, these estimates are likely conservative, as misdiagnosis can result in a high degree of underreporting^2^.

Dengue is transmitted to and between humans by infected female mosquitoes, primarily through *Aedes aegypti*, although *Ae. albopictus* is also a recognized secondary vector. The disease is caused by any of the four serologically distinct yet antigenically-related dengue virus (DENV) serotypes (DENV-1, DENV-2, DENV-3, and DENV-4)^6^. Infection by one of the serotypes induces long-term homotypic protection and transient heterotypic protection, lasting up to 2 years on average^7^.

There is no specific treatment for dengue, and control of the disease relies mainly on the improvement of hygiene measures, using personal or household protection from vector exposure, or applying insecticides against the main vectors, in order to reduce transmission. Recently, vaccination against DENV became a viable alternative prevention measure in certain populations, as the live-attenuated recombinant tetravalent vaccine employing an attenuated strain of the yellow fever virus (CYD-TDV; *Dengvaxia*, Sanofi Pasteur) has been licensed in several countries^8^. However, during the vaccine’s clinical development, an increased incidence of hospitalizations and severe dengue was noted in initially dengue-seronegative individuals, following vaccination with CYD-TDV in phase 3 trials^9^. Therefore, its use is recommended by the World Health Organization only in dengue-seropositive individuals of at least nine years of age^8^. Several other investigational vaccines are under development, having reached phase 2 or 3 trials^10,11^.

An investigational tetravalent dengue purified inactivated vaccine (DPIV) consisting of DENV-1, -2, -3, and -4 strains produced in Vero cells and inactivated with formalin was also developed, and its safety and immunogenicity were evaluated in adults in two previous phase I studies^12,13^. Encouraging safety and immunogenicity data were reported in both predominantly dengue-naive^12^ or predominantly dengue-primed healthy adults^13^ who received two doses of four different formulations of DPIV with three different adjuvants: 1 μg each of DENV-1, -2, -3, and -4 adjuvanted with alum (Alhydrogel); 1 μg each of DENV-1, -2, -3, and -4 adjuvanted with AS01_E_; 1 μg each of DENV-1, -2, -3, and -4 adjuvanted with AS03_B_; or 4 μg each of DENV-1, -2, -3, and -4 adjuvanted with alum. A placebo group received saline as a control. Safety data and immune responses to DENV-1–4 as evaluated by microneutralization assay were also reported in a long-term follow-up study^14^. Here, we report results of the exploratory objectives from these studies, which were to evaluate cell-mediated immunity (CMI) elicited by each of the four DPIV formulations in terms of antigen-specific memory B-cell and T-cell responses. In addition, B-cell and T-cell responses were evaluated in a subset of baseline dengue-naive adult participants through 1 month following the administration of a booster dose of DPIV one year after the initial 2-dose regimen.

## Results

### According-to-protocol (ATP) study cohort

The demographic characteristics of participants from both studies were previously reported^12,13^. Briefly, in each study, 100 participants were enrolled and randomized (1:1:1:1:1) to receive either 1 μg per DENV-1–4 adjuvanted with either alum, AS01_E_, or AS03_B_, or 4 μg per DENV-1–4 adjuvanted with alum, or placebo. The adapted ATP cohorts in which the analyses of B-cell and T-cell responses were performed included 13–19 participants per group. Nine initially dengue-naive adult participants (three participants from the 4 μg + Alum group and six participants from the 1 μg + AS01_E_ group) were vaccinated with a booster dose of the same formulation as the initial vaccination^12^.

### B-cell responses

**Fig. 1:**
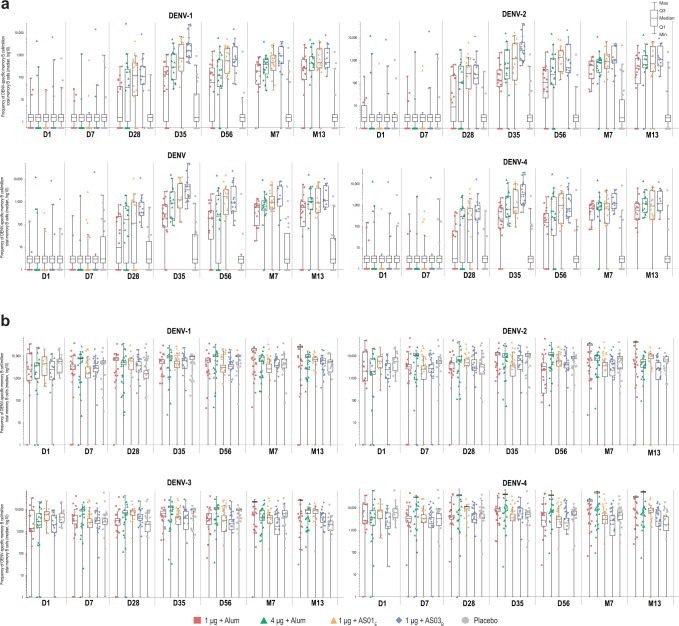
Frequencies of memory B cells specific to dengue virus serotypes 1–4. Values are shown at multiple time points in dengue-naive (**a**) and dengue-primed (**b**) adults following 2-dose DPIV vaccination (adapted according-to-protocol cohort for immunogenicity). Error bars on the box plot represent the Min and Max values in each group. Min and Max values were calculated as (Q1–1.5*interquartile range)/(Q3 + 1.5* interquartile range). Individual values are plotted for each group. D, day; D0, pre-vaccination; D7, 7 days post-dose 1; D28, 28 days post-dose 1; D35, 7 days post-dose 2; D56, 28 days post-dose 2; DENV, dengue virus; DPIV, tetravalent dengue purified inactivated vaccine; M, month; M7, 6 months post-dose 2; M13, 12 months post-dose 2.

Among dengue-naive adults, no DENV-specific memory B cells were detected by enzyme-linked immunospot (ELISPOT) assay pre-vaccination. Memory B cells specific to each DENV serotype were detectable after the 2-dose series and persisted up to month 13. Overall, in participants receiving each of the four DPIV formulations, responses peaked at 7 days post-dose 2 (day 35 after initiation of vaccination) and remained higher than at pre-vaccination for the entire follow-up time. There was a trend for higher responses to the 1 μg + AS01_E_ and 1 μg + AS03_B_ formulations compared with the alum-adjuvanted formulations up to month 7 (Fig. 1a). At month 13, median frequencies of DENV-specific memory B cells per million total memory B cells varied from 480 to 860 for DENV-1, from 435 to 1069 for DENV-2, from 548 to 1114 for DENV-3, and from 578 to 1177 for DENV-4. No response was observed in placebo recipients.

Among dengue-primed adults, the median frequency of DENV-specific memory B cells per million total memory B cells ranged between 1292 and 7216 across all DENV serotypes and study groups at pre-vaccination. Following two doses of DPIV (day 35 of the study), the frequency of memory B cells specific to each DENV serotype increased in all vaccinated groups (Fig. 1b). At month 13, in vaccinated individuals, median frequencies of DENV-specific memory B cells per million total memory B cells varied from 2251 to 5425 for DENV-1, from 1413 to 6874 for DENV-2, from 1037 to 5526 for DENV-3, and from 2440 to 7944 for DENV-4.

Overall, and as expected, the frequency of memory B cells prior to vaccination was substantially higher in the dengue-primed than in the dengue-naive adults. The higher frequency of memory B cells in dengue-primed versus dengue-naive adults was maintained post-vaccination through month 13 for all four DENV serotypes (Fig. 1).

**Fig. 2:**
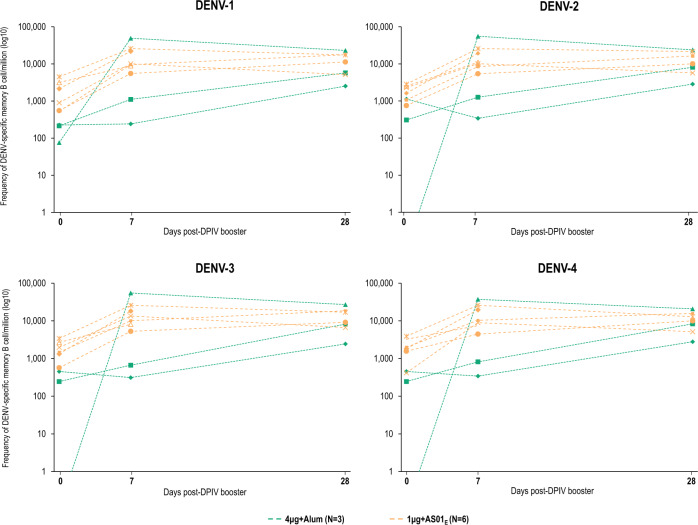
Individual frequencies of memory B cells specific to dengue virus serotypes 1–4. Values shown are for a subset of dengue-naive participants who received a DPIV booster (according-to-protocol cohort for immunogenicity for dose 3 subset). DENV, dengue virus; DPIV, tetravalent dengue purified inactivated vaccine; N, number of study participants who received a DPIV booster. Lines indicate responses of individual participants.

A booster dose given 15–21 months after priming with two doses of DPIV in a small subset of initially dengue-naive adults induced strong anamnestic memory B-cell responses (Fig. 2). All nine participants given a booster dose showed increased DENV-specific memory B-cell frequencies up to 28 days post-booster dose (the last time point tested), when median frequencies of DENV-specific memory B cells per million total memory B cells were 5720 and 14,408 for DENV-1, 8110 and 13,230 for DENV-2, 8182 and 13,031 for DENV-3, and 8317 and 11,419 for DENV-4 in 4 μg + Alum and 1 μg + AS01_E_ recipients, respectively.

### T-cell responses

**Fig. 3:**
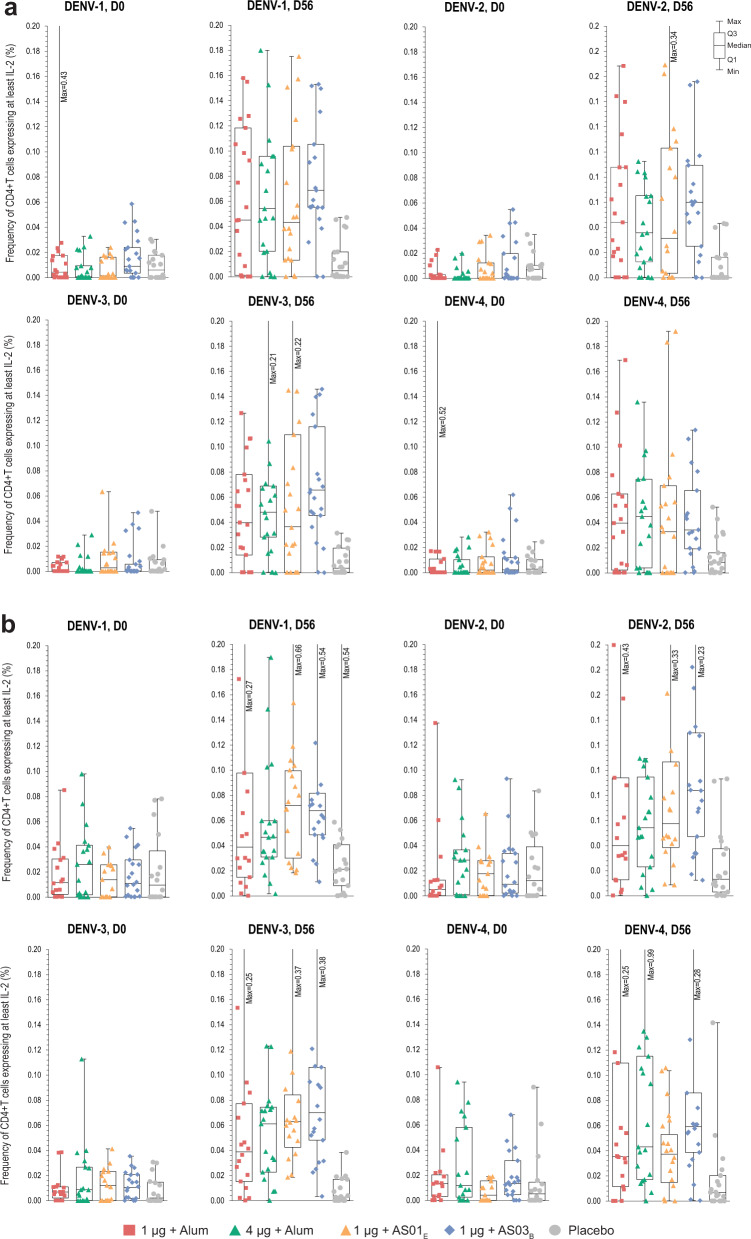
Frequencies of CD4+ T cells specific to dengue virus serotypes 1–4 expressing at least IL-2. T cells were quantified using the combined capsid/pre-membrane and envelope region peptide pools in the predominantly dengue-naive (**a**) and dengue-primed (**b**) populations (according-to-protocol cohort for cell-mediated immunogenicity at day 56), at day 0 (pre-vaccination) and day 56 following 2-dose DPIV administration. Individual values are plotted for each group. Error bars on the box plot represent the Min and Max values in each group. D, day; D0, pre-vaccination; D56, 28 days post-dose 2; DENV, dengue virus; DPIV, tetravalent dengue purified inactivated vaccine; Min/Max, minimum/maximum observation; Q1, Q3, first and third quartiles; ◊, geometric mean frequency. For each serotype, the combined capsid/pre-membrane and envelope region peptide pools ((C + M) + E pool)-specific frequency was derived by adding the (C + M) pool specific frequency and the E pool specific frequency. Note: Data were not background-subtracted.

CD4+ and CD8+ T cells expressing at least two functional markers among CD154 (also called CD40 ligand [CD40L]), interleukin (IL)-2, tumor necrosis factor (TNF)-α, interferon (IFN)-γ, CD107a, and macrophage inflammatory protein (MIP)-1β were quantified pre-vaccination and at day 56 (28 days post-dose 2) using overlapping peptide pools representing the combined capsid/pre-membrane and envelope (CME) regions of each DENV serotype. The intracellular cytokine staining (ICS) gating strategy used for this analysis is shown in Supplementary Fig. 1. DPIV vaccination induced a DENV-specific multifunctional CD4+ T-cell response, with IL-2 as the most important component (Fig. 3 and Supplementary Fig. 2). The cytokines most frequently co-expressed with IL-2 were IFN-γ and TNF-α. In dengue-naive adults, the median frequency of DENV-specific CD4+ T cells expressing at least IL-2 varied from 0.04% to 0.07% for DENV-1, from 0.03% to 0.06% for DENV-2, from 0.04% to 0.07% to DENV-3, and from 0.03% to 0.04% for DENV-4, among the four DPIV formulations at day 56 (Fig. 3a). In dengue-primed adults, DENV-specific CD4+ T cells expressing at least IL-2 were slightly higher at baseline compared to naive adults but also increased in frequency post-vaccination (Fig. 3b). At day 56, the median frequency of DENV-specific CD4+ T cells expressing at least IL-2 varied from 0.04% to 0.07% for DENV-1, from 0.04% to 0.08% for DENV-2, from 0.04% to 0.07% for DENV-3, and from 0.04% to 0.06% for DENV-4, among the four DPIV formulations. Similar to those observed in the dengue-naive population, these responses were multifunctional (Supplementary Fig. 3).

In both populations (naive and dengue-primed) there were numerically higher T-cell responses in the 1 μg + AS03_B_ group, but the sample size and requirement for multiple comparisons did not permit a formal post hoc statistical analysis among the DPIV formulations. No change from pre-vaccination CD4+ T-cell frequencies were observed in the groups receiving placebo for any of the four DENV serotypes in either population. No vaccine-induced DENV-specific CD8+ T-cell responses were detected in the dengue-naive adults. In the dengue-primed adults, low levels of DENV-specific CD8+ T cells were detectable pre-vaccination and did not change after DPIV vaccination (Supplementary Fig. 4).

DENV-specific CD4+ T cells expressing at least IL-2 were also detected following the administration of a booster dose of 1 μg + AS01_E_ or 4 μg + Alum in the subset of initially dengue-naive participants, showing an increase for all four DENV serotypes at day 28 (Supplementary Fig. 5).

**Fig. 4:**
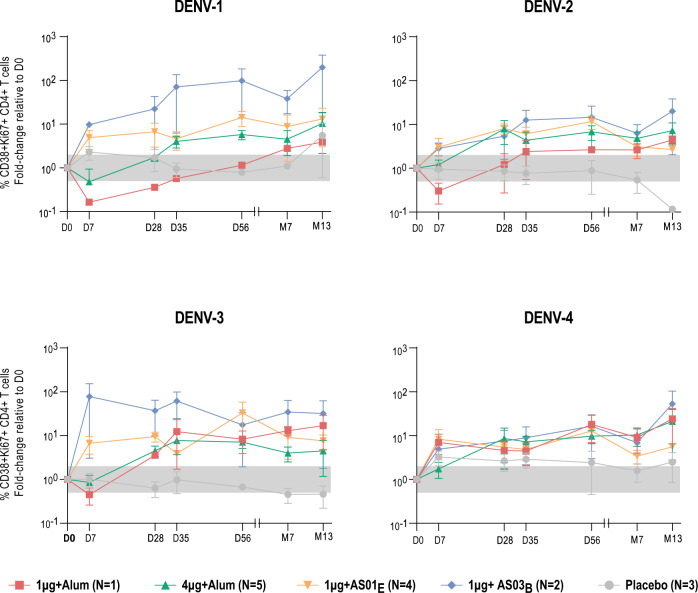
Median fold change in the percentage of activated CD38 + Ki67 + CD4+ T cells among total CD4+ T cells. Changes are relative to day 0 (pre-vaccination) values, after 6–7 days in vitro culture with combined DENV capsid/pre-membrane and envelope region peptide pools, following DPIV vaccination of dengue-naive adults (adapted according-to-protocol cohort for immunogenicity subset). Error bars represent standard errors. D, day; D0, pre-vaccination; D7, 7 days post-dose 1; D28, 28 days post-dose 1; D35, 7 days post-dose 2; D56, 28 days post-dose 2; DENV, dengue virus; DPIV, tetravalent dengue purified inactivated vaccine; M, month; M7, 6 months post-dose 2; M13, 12 months post-dose 2; N, number of participants, expressed as a range. The gray box indicates +/− two-fold.

**Fig. 5:**
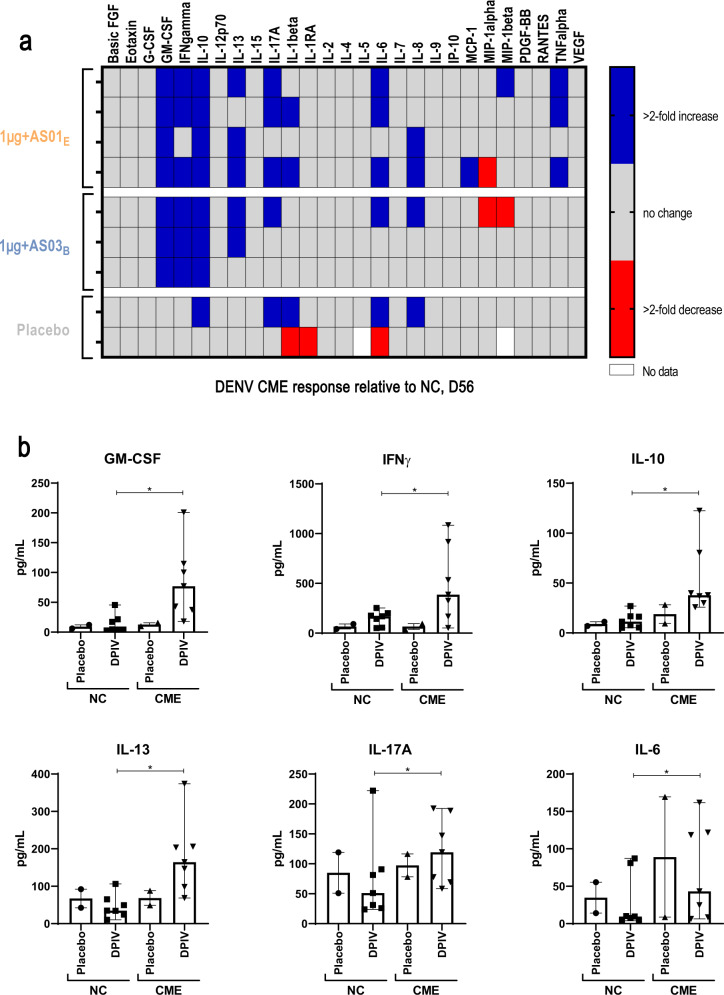
Cytokine analysis of 6–7 days culture supernatants. The panels show the difference in the production of 27 cytokines (using combined DENV capsid/pre-membrane and envelope region peptide pools) at day 56 (**a**) and the concentrations of cytokines showing a greater than two-fold change in at least four vaccinated individuals (**b**) (adapted according-to-protocol cohort for immunogenicity subset). Error bars represent the Min and Max values in each group. CME, combined capsid/pre-membrane and envelope; D, day; D56, 28 days post-dose 2; DENV, dengue virus; FGF, fibroblast growth factor; G-CSF, granulocyte colony-stimulating factor; GM-CSF, granulocyte-macrophage colony-stimulating factor; IFN, interferon; IL, interleukin; IP-10, interferon-inducible protein 10; MCP-1, monocyte chemoattractant protein-1; MIP, macrophage inflammatory protein; NC, negative control; PDGF, platelet-derived growth factor; TNF, tumor necrosis factor; VEGF, vascular endothelial growth factor. *Significant difference in the DPIV groups comparing NC vs CME stimulation as assessed by Holm-Sidak using *α* = 0.05; computations assumed that all time points were sampled from populations with the same scatter.

In a subset of dengue-naive participants, pre-vaccination peripheral blood mononuclear cell (PBMC) samples and those taken at multiple time points post-vaccination were placed in short-term culture with DENV CME peptide pools in order to increase the sensitivity of detecting antigen-specific T cells. After ~6–7 days, DENV-reactive cells were identified by flow cytometry based on the expression of activation markers (Supplementary Fig. 6). The frequency of DENV-reactive CD38 + Ki67 + CD4 + T cells increased from pre-vaccination in all groups receiving DPIV formulations, as compared to those receiving placebo, particularly after the second dose of vaccine and continuing through month 13 of the study (Fig. 4; Supplementary Table 1). Cell culture supernatants harvested during the proliferation experiments performed above were assessed for the presence of a large panel of cytokines. Supernatants from nine dengue-naive adults (four, three, and two participants in the 1 μg + AS01_E_, 1 μg + AS03_B_, and placebo groups, respectively) demonstrated that DPIV vaccination increased antigen-specific production of granulocyte-macrophage colony-stimulating factor (GM-CSF), IFN-γ, IL-10, IL-13, IL-17A, and IL-6 (Fig. 5).

## Discussion

Studies with DPIV have demonstrated a very good safety and tolerability profile^12,13,15^. Immunogenicity assessments in terms of neutralizing antibody have shown that DPIV induces robust and balanced tetravalent peak titers. However, these titers waned quickly, particularly in dengue-naive individuals^12,13^. Herein, we sought to further characterize the immunogenicity of this investigational vaccine in terms of CMI. The exploratory analyses presented here show that all four evaluated DPIV formulations induced durable DENV-specific memory B-cell and CD4 + T-cell responses, which generally appeared strongest for the AS01_E_- and AS03_B_-adjuvanted formulations. CMI responses were observed following vaccination with any DPIV formulation, regardless of the vaccinees’ initial DENV immune status. However, the frequencies of memory B cells were generally higher in the dengue-primed healthy adults compared to the dengue-naive adults, which is consistent with their pre-existing immunity due to prior natural infection. T-cell responses were predictably CD4+ in phenotype, and while low in frequency, they were readily detectable in most participants regardless of initial DENV immune status.

The observed durability of memory B-cell responses in the periphery is an interesting finding when compared with previously reported DENV-specific neutralizing antibody responses^12,13^. This indicates that at least in some individuals, DPIV induces a robust memory B-cell response that persists despite waning neutralizing antibodies; Supplementary Fig. 7 presents this trend as observed in the current study in the dengue-naive population. Since antibody levels are maintained by long-lived plasma cells (most likely in the bone marrow), there appears to be a bias in the ability of this vaccine’s strategy to generate effective circulating memory B-cell responses and only short-lived plasma cell responses. Notably, none of the four different adjuvant formulations modulated this apparent bias. These data stand in contrast to the long-lasting (up to 40 weeks post-immunization) circulating antibody responses induced by DPIV in rhesus macaques^16^. This difference between persistent neutralizing antibodies in rhesus macaques and waning neutralizing antibodies in humans may reflect species-specific differences in response to the antigen and adjuvant formulations administered. Regardless, the phenomenon of the generation and maintenance of B-cell memory in the absence of durable neutralizing antibodies and plasma cells is important and requires further study.

In the small subset of dengue-naive participants who received a DPIV booster dose, strong anamnestic B-cell responses were induced, underscoring the ability of DPIV, and notably the AS01_E_-adjuvanted formulation, to readily boost an existing memory B-cell response. The third dose also robustly boosted neutralizing antibody titers^12^. Similarly, in a previous study, administration of the AS01_B_-adjuvanted formulation according to a 0-, 1-, and 6-month schedule induced higher peak neutralizing antibody response than 2-dose schedules at 1-month post-last vaccination. However, after 12 months, similar memory B cells frequencies and neutralizing antibody levels were observed for the 0-, 1-, and 6-month and the 0-, and 3-month schedules^15^. A limitation of the memory B-cell ELISPOT assay employed here is that it does not distinguish DENV serotype-specific versus DENV serotype-cross-reactive memory B cells. Future studies utilizing assays that have recently been published to evaluate DENV serotype specificity of DPIV-induced memory B cells are warranted^17^.

All DPIV formulations elicited IL-2-expressing multifunctional CD4+ T cells against all four DENV serotypes in both dengue-primed and dengue-naive individuals. The observed disparity between detection of IL-2-producing cells by ICS and lack of IL-2 detection in the cell culture supernatants is likely attributable to IL-2 “consumption” by the proliferating T cells in the 7-day cultures. The most common co-expressed cytokines with IL-2 were IFN-γ and TNF-α, indicating a T helper 1 (Th1) response. This assertion is further corroborated by the detection of GM-CSF and IFN-γ in the supernatants of in vitro DENV antigen-stimulated PBMCs in dengue-naive adults. While an in-depth study of the quality of the CD4+ T-cell response was not undertaken here, the ability of these cells to activate, proliferate, and produce immunologically relevant cytokines post-vaccination suggests that DPIV is able to induce low level yet robust and durable CD4+ T-cell responses. Furthermore, the increase in frequency of DENV-specific CD4+ T cells after the administration of a third dose of DPIV during the second year after initial vaccination demonstrates that these cells are responsive in vivo to booster vaccination. The lack of detectable vaccine-induced, antigen-specific CD8+ T cells is not surprising, given that inactivated vaccines in general rarely induce appreciable CD8+ T-cell responses^18,19^ and that the majority of DENV CD8+ epitopes are located in the non-structural proteins^20^. Similarly to DPIV, CYD-TDV (the only currently licensed vaccine for dengue), presents only the structural components of DENV to the immune system. DENV-specific T-cell responses generated by CYD-TDV are also predominantly CD4+ and of a Th1 phenotype^21^. However, little data exist on the precise frequency of the vaccine-elicited response^22–24^. Furthermore, CYD-TDV-elicited CD8+ T cells appear to be restricted to non-structural components of the vaccine derived from yellow fever virus, and are poorly cross-reactive with DENV^21^. When reconciled with our prior study assessing the CMI generated in response to a monovalent PIV (DENV-1)^25^, we conclude that formalin-inactivated PIVs induce reproducibly, detectable, and durable, albeit low frequency CD4+ T cells capable of responding to recall antigen in vitro and in vivo.

This study aims at evaluating both B-cell and T-cell responses elicited by DPIV, or any PIV against dengue, in a clinical setting. In summary, these data on B- and T-cell responses induced by DPIV administration complement the observed robust humoral immunogenicity of the vaccine in dengue-primed adults^13^. However, the promise of DPIV was that it would induce an immune response in dengue-naive individuals that is tetravalent, balanced, and durable—three important features that are critical requirements for a dengue vaccine^26^. While published studies have confirmed that the neutralizing antibody response generated by DPIV is tetravalent and balanced^12,15^, a concern raised by these same studies is that the neutralizing antibody response demonstrated rapid waning and poor durability in dengue-naive adults. The data presented here show that durable B and T-cell immunity is established following DPIV vaccination in dengue-naive adults^12^ and can be boosted in previously vaccinated and dengue seropositive adults. Therefore, future development of related vaccines should addess the dislinkage between B-cell and T-cell memory and longevity of circulating neutralizing antibodies.

## Methods

### Study design

Both studies were phase I, randomized, placebo-controlled, and observer blind. The design, inclusion/exclusion criteria and primary objectives for both trials have been previously described in detail^12,13^. Briefly, the study in the predominantly dengue-naive population (NCT01666652) was conducted at the Clinical Trials Center at the Walter Reed Army Institute of Research (WRAIR), Silver Spring, Maryland, United States and enrolled healthy male and female adults between 18 and 39 years of age. The second trial (NCT01702857) was conducted at the University of Puerto Rico Medical Sciences Campus, Puerto Rico Clinical and Translational Research Consortium Center, San Juan, Puerto Rico, United States in predominantly dengue-primed healthy male and female adults aged 20–39 years, who had lived in the Caribbean for more than 10 years. Immunogencitiy analyses were restricted to dengue-naive and dengue-primed individuals in the first and second trial, respectively. In both trials, participants were randomized 1:1:1:1:1 to receive two doses of different formulations of DPIV, administered four weeks apart (on days 0 and 28): 1 μg/serotype/dose adjuvanted with either alum (Group 1 μg + Alum), AS01_E_ (Group 1 μg + AS01_E_), or AS03_B_ (Group 1 μg + AS03_B_), 4 μg/serotype/dose adjuvanted with alum (Group 4 μg + Alum), or placebo. AS01_E_ is an adjuvant system containing 3-*O*-desacyl-4’-monophosphoryl lipid A (MPL; GSK), QS-21 (*Quillaja saponaria* Molina, fraction 21) (licensed by GSK from Antigenics LLC, a wholly owned subsidiary of Agenus, Inc., a Delaware, United States corporation), and liposome (25 μg MPL and 25 μg QS-21). AS03_B_ is an adjuvant system containing DL-α-tocopherol and squalene in an o/w emulsion (5.93 mg DL-α-tocopherol). A subset of nine participants in the NCT01666652 trial also received a third (booster) dose of DPIV, at 15–21 months after the second vaccine dose^12^. The vaccine composition was previously described in detail^12^.

Exploratory objectives, presented here, evaluated the CMI responses induced by the four DPIV formulations at days 0 (pre-vaccination), 7 (7 days post-dose 1), 28 (28 days post-dose 1/pre-dose 2), 35 (7 days post-dose 2), and 56 (28 days post-dose 2) and months 7 (6 months post-dose 2) and 13 (12 months post-dose 2) for B-cell responses and at days 0 and 56 for T-cell responses. The proliferative and cytokine-producing capacity of DPIV-elicited T cells were evaluated at all time points in a subset of dengue-naive adults. B-cell responses prior to and following a DPIV booster dose (pre-booster and at 7 and 28 days post-booster) and T-cell responses (pre-booster and at 28 days post-booster) were also evaluated in a subset of participants. At each time point, ~40 mL of blood were collected from participants in each subset.

Both studies were conducted in agreement with the Declaration of Helsinki, International Conference on Harmonization and Good Clinical Practice Principles, and their protocols and associated documents were approved by institutional review boards (NCT01666652: WRAIR Institutional Review Board, the Office of Research Protections, Human Research Protection Office, the U.S. Army Medical Materiel Development Activity, and GSK; NCT01702857: U.S. Army Human Subjects Research Review Board, Office of the Surgeon General, the U.S. Army Medical Materiel Development Activity, GSK, and the Western Institutional Review Board on behalf of the University of Puerto Rico). Written and signed informed consent to participate were obtained from eligible individuals or their legally authorized representative.

### Immunogenicity assessments

#### DENV serotype-specific memory B-cell responses

The frequency of DENV serotype-specific antibody-secreting cells (ASCs) per million ASCs was determined using an adapted ELISPOT method developed by Crotty et al.^27^. Briefly, the method consisted of a first incubation of PBMCs during 5 days at 37 °C and 5% CO_2_ in presence of cytosine phosphate guanine (CpG 2006 – 1 µg/mL; prepared in RPMI-1640 medium supplemented with additives and 10% heat-inactivated fetal bovine serum) for differentiation into memory B cells. In parallel, polyvinylidene difluoride plates were coated with either the antigens of interest (inactivated DENV-1–4 virions; coated at 5 µg/ml; prepared in Dulbecco’s phosphate-buffered saline [DPBS]), host-cell protein (control condition: cells lysate coated at 5 µg/mL, prepared in DPBS) or anti-human immunoglobulin G (IgG) (Affinipure Goat anti-human IgG ‘H + L’ from Jackson Laboratories; coated at 50 µg/mL; prepared in DPBS). Coated plates were stored at 4 °C. At day 5, washed and counted cells were loaded at concentration of 200,000 cells per specific antigens and control condition well (in triplicates) or at concentration of 2000 cells per IgG well (in triplicates). After overnight incubation (37 °C - 5% CO_2_), cell removal and washing steps, spot forming cells (SFCs) are revealed by the addition of a secondary antibody (biotynilated IgG; two hours at room temperature at concentration of 2 µg/mL; prepared in saturated buffer: DPBS + bovine serum albumin 10%+ fetal bovine serum 4%), addition of a conjugate complex (extravidin peroxydase; one hour at room temperature at concentration of 4 µg/mL; prepared in saturated buffer; after washing steps), and addition of 3 amino-9 ethyl-carbazole (10 minutes at room temperature, protected from light; after washing steps). After last washing steps and drying of plates overnight at room temperature, SFCs were counted by the ELISPOT plate reader. The results were expressed as the frequencies of antigen-specific memory B cells within the total memory B-cell population (proportion of antigen-specific memory B cells per million total memory B cells).

#### Peptides for T-cell stimulation assays

Pools of 12–20-mer-peptides overlapping by 10–12 amino acids and corresponding to the full-length E proteins of DENV-1 Nauru/West Pac/1974, DENV-2 New Guinea C, DENV-3 Philippines/H87/1956, and DENV-4 Singapore/8976/1995 were obtained from BEI Resources. Peptide pools (16 mers overlapping by 11 amino acids) covering both the C and M proteins of DENV-1–4 were purchased from JPT Peptide Technologies. Peptide pool stocks were reconstituted in 100% dimethyl sulfoxide (DMSO) at a concentration of 200 μg/mL/peptide and stored at –80 °C.

#### ICS assay

Cryopreserved PBMCs were thawed and 1 × 10^6^ PBMC were plated per well of a 96-well plate in a total volume of 200 μL of R10 medium (RPMI-1640 medium supplemented with 10% fetal bovine serum, L-glutamine, penicillin, and streptomycin) along with anti-CD28, anti-CD49d, and fluorescein isothiocyanate-conjugated anti-CD107a antibodies (BD Biosciences) and 1 μg/mL/peptide of the relevant peptide pool. R10 containing 0.5% DMSO was used as a negative control, and a mixture of 50 ng/mL phorbol 12-myristate 13-acetate and 1 μg/mL ionomycin was used as a positive control. Cells were incubated at 37 °C for one hour prior to addition of brefeldin A and monensin (BD Biosciences) and then left to continue incubating overnight. The next day, cells were washed and stained with LIVE/DEAD Aqua (Invitrogen, Life Technologies, Thermo Fisher Scientific) followed by the surface antibodies Brilliant Violet 785-conjugated anti-CD3 (BV785-CD3), BV605-CD4, BV650-CD8 (BioLegend), Alexa700-CD14, and Alexa700-CD19 (BD Biosciences). After fixation in 4% formaldehyde, cells were permeabilized and stained with the antibodies eFluor450-IFN-γ, PE-Cy7-TNFα, PE-MIP-1β, APC-IL-2, and PE-Cy5-CD154 (BD Biosciences). Data were collected using a BD LSRFortessa flow cytometer (BD Biosciences).

#### Short-term in vitro culture

Cryopreserved PBMCs were thawed and plated at ~1 × 10^6^ cells per well in R10 medium with 0.5 μg/mL/peptide of the respective DENV-1–4 CME peptide pool. R10 containing 0.5% DMSO was used as a negative control. Cells were cultured at 37 °C for 6–7 days, and the supernatants were stored at –80 °C for the cytokine analysis. The cells were washed and stained with LIVE/DEAD Aqua as well as BV785-CD3, BV605-CD4, Alexa700-CD8, BV510-CD14, BV510-CD19, APC-Cy7-CD16, PE-Cy7-CD56, PE-CD38, and PE-Dazzle594-HLA-DR (BD Biosciences). After fixation in 4% formaldehyde, cells were permeabilized and stained with Alexa488-Ki67 (BD Biosciences). Data were collected using a BD LSRFortessa flow cytometer (BD Biosciences).

#### Cytokine analysis

Short-term cell culture supernatants were thawed and tested (in duplicate, undiluted) using the Bio-Plex Pro™ Human Cytokine 27-plex kit (M500KCAF0Y, Bio-Rad) following the manufacturer’s instructions. Protein standards were provided in the kit, and standard curves were generated with eight standard dilutions (undiluted, 1:3, 1:9, 1:27, 1:81, 1:243, 1:729, 1:2187) using a five-parameter logistic curve fit and 1/y^2^ weighted function. Data were acquired on a MAGPIX instrument (Bio-Rad).

### Statistical analysis

Considerations regarding the sample size and power calculations were previously reported in detail for each study^12,13^.

At each time point, the evaluation of CMI was performed in the adapted ATP cohort for immunogenicity, including all evaluable participants who had data available for the evaluated immunogenicity endpoint at each time point. In dengue-naive adults, the ATP immunogenicity cohort at day 56 was used for all time points up to day 56, and the ATP immunogenicity cohort at month 7/13 was used for month 7/13. In dengue-primed adults, the adapted ATP cohort for immunogenicity included all participants from the day 56 analysis who had blood samples available for immunogenicity testing.

The frequencies of DENV serotype-specific CD4+ or CD8+ T cells were calculated as the difference between the frequency of CD4+ or CD8+ T cells expressing immune markers upon in vitro stimulation with DENV antigen minus the frequency of CD4+ or CD8+ T cells expressing these immune markers upon in vitro stimulation with the negative control (medium plus 0.5% DMSO), called background. Differences less than or equal to zero were imputed to one antigen-specific immune marker-expressing CD4+ or CD8+ T cell per million CD4+ or CD8+ T cells.

Data were analyzed using software packages: FlowJo (Becton, Dickinson & Company); Microsoft Excel (Microsoft Corp.); and GraphPad Prism (GraphPad Software).

All analyses were descriptive.

### Reporting summary

Further information on research design is available in the Nature Research Reporting Summary linked to this article.

## Supplementary information

Supplemental material

REPORTING SUMMARY

## Data Availability

Information on GSK’s data sharing commitments and requesting access to anonymized individual participant data and associated documents from GSK sponsored studies can be found at www.clinicalstudydatarequest.com (study ID: 116289/116614). To access data for other types of GSK sponsored studies, visit www.clinicalstudydatarequest.com. Questions regarding information on WRAIR data sharing commitments and access can be directed to the WRAIR Research Programs Office.
