# Supplementary material for: Characterization of B-cell and T-cell responses to a tetravalent dengue purified inactivated vaccine in healthy adults
Source: NPJ Vaccines. 2022 Oct 31;7:132. doi: 10.1038/s41541-022-00537-2 (PMC9622737; doi:10.1038/s41541-022-00537-2)
Supplement: Supplementary file 1 — Supplemental material [file 41541_2022_537_MOESM1_ESM.pdf]

Title page

Manuscript title: Characterization of B-cell and T-cell responses to a tetravalent dengue purified inactivated vaccine in healthy adults

Author(s): Heather Friberg[1,*], Morgan Gargulak[1], Amanda Kong[1], Leyi Lin[1], Luis J. Martinez[1], Alexander C. Schmidt[2], Robert M. Paris[2], Richard G. Jarman[1], Clemente Diaz[3], Stephen J. Thomas[1], Philippe Moris[4] and Jeffrey R. Currier[1]

Affiliations:

[1]Viral Diseases Branch, Walter Reed Army Institute of Research, Silver Spring, MD, United States

[2]GSK, Rockville, MD, United States

[3]University of Puerto Rico School of Medicine, San Juan, Puerto Rico

[4]GSK, Rixensart, Belgium

Corresponding author:

*Heather Friberg

Walter Reed Army Institute of Research

Silver Spring, MD, United States.

heather.l.friberg-robertson.civ@mail.mil

Supplementary table 1. Fold change in the percentage of activated CD38+ Ki67+ CD4+ T cells among total CD4+ T cells for dengue-naïve adults (adapted according-to-protocol cohort for immunogenicity subset)

	1 µg + Alum			4 µg + Alum					1 µg + AS01$_E$				1 µg + AS03$_B$				Placebo			
	P1	P2	P3	P1	P2	P3	P4	P5	P1	P2	P3	P4	P1	P2	P3	P4	P1	P2	P3	P4
DENV-1																				
D7	0.164634			2.297297	0	0.110931	0	0	7.757764	6.441176	0.502513		11.12766		8.333333		1.5	3.086957		
D28	0.359756			2.306306	2.348624	0.778138	2.077778	1.144737	4.459627	17.79412	2.130653	2.436916	1.468085		43.16667		0.518433	3.478261	1.169725	
D35	0.567073			2.576577	7.247706	0.926316	6.222222	3.144737	1.993789	10.76471	3.100503	2.567757	6.238298		135.6667		0.875576	1.565217	0.385321	
D56	1.152439			6.846847	5.431193	0.617814	8.722222	7.171053	19.68944	19.5	3.427136		13.97872		182.3333		0.698157		0.880734	
M7	2.792683			2.657658	2.541284	0.652632	1.888889	14.78947	2.826087	23.52941		0.079439	17.34043		59		1.170507		1.027523	
M13	3.890244			1.54955	2.504587	0.845344	3.677778	43.13158	3.037267	23.23529			18.19149		378.3333			10.47826	0.59633	
DENV-2																				
D7	0.456311	0.151515		1.160752	0.682927	1.785714			1.568627	0.942466	6.591837		1.420664	4.232456	4.583333	1.08744	1.449561		0.150696	1.25061
D28	0.271845	2.151515		0.444676	21.56098	14.875	1.109589	1.235577	6.45098	2.882192	13.5102	9.267123	0.350554	2.776316	16.66667	1.798092	1.530702		0.443079	0.59213
D35	0.553398	4.280303		1.039666	4.97561	13.17857	1.029354	1.519231	5.245098	2.353425	10.7551		4.971218	6.171053	37.41667	1.761526	1.342105		0.150696	0.80098
D56	2.291262	3.007576		1.480167	10.41463	14.33929	1.780822	5.956731	14.41176	3.816438	16.57143		4.084871		37.75	1.736089	2.133772		0.289107	0.21867
M7	3.582524	1.659091		1.757829	4.487805	11.26786	0.489237	5.980769	5.343137	2.767123		1.10274	4.00369	3.618421	16.75	0.82194	1.063596		0.410319	0.14496
M13	5.446602	3.378788		0.340292	3.04878	16.67857	0.618395	15.375	2.823529	2.528767			3.450185		56	0.6407			0.107289	0.12530
DENV-3																				
D7	0.638298	0.26087		1.616541	0.375	1.402985	0.013793		9.617021	3.75188			3.006431	5.870229	7.733333	3.063232	1.27809	0.986111	0.031164	1.74911
D28	3.170213	3.956522		1.132832	6.4375	6.328358	4.193103		11.93617	7.082707			0.324759	2.793893	24.66667	2.320843	0.210674	0.444444	0.538038	1.33922
D35	1.702128	23.08696		1.473684	5.0625	19.76119	4.731034		4.723404	3.766917		3.24375	0.875241	2.122137	29.46667	3.274005	0.606742	0.736111	0.12374	2.44876
D56	3.893617	12.73913		1.974937	10.21875	10.35821	5.82069		58.29787	7.842105			2.209003		43.2	3.775176	0.866573		0.505041	0.65371
M7	9.87234	16.08696		1.443609	3.125	8.283582	3.089655		21.59574	4.518797		1.39375	4.292605	2.984733	13.46667		0.549157	0.074074	0.324473	0.86925
M13	29.19149	4.73913		0.842105		11.1194	1.510345		10.34043	4.894737			2.78135		103.3333			0.953704	0.197984	0.24028
DENV-4																				
D7	0.907692	6.116279	13.96	4.422222	1.136364	1.144068	0.45045	1.5625	19.08989	1.688525	4.06		3.006431	5.870229	7.733333	3.063232	0.375	1.402985	0.013793	0.03526

	1 µg + Alum			4 µg + Alum					1 µg + $AS01_E$				1 µg + $AS03_B$				Placebo			
	**P1**	**P2**	**P3**	**P1**	**P2**	**P3**	**P4**	**P5**	**P1**	**P2**	**P3**	**P4**	**P1**	**P2**	**P3**	**P4**	**P1**	**P2**	**P3**	**P4**
D28	0.876923	2.55814	10.3	0.24	33.36364	3.101695	2.414414	4.03125	6.52809	2.557377	9.1	3.794118	0.324759	2.793893	24.66667	2.320843	6.4375	6.328358	4.193103	0.29237
D35	0.661538	8.55814	4.14	0.488889	9.045455	4.084746	7.252252	15.15625	1.05618	2.245902	10.7	5.347059	0.875241	2.122137	29.46667	3.274005	5.0625	19.76119	4.731034	0.52332
D56	2.323077	12.30233	39.48	5.466667	13.18182	4.974576	4.189189	20.84375	23.93258	2.770492	11.94		2.209003		43.2	3.775176	10.21875	10.35821	5.82069	0.62229
M7	2.323077	5.953488	19.06	8.364444	6.954545	4.228814	3.504505	28.5625	5.752809	1.95082		2.523529	4.292605	2.984733	13.46667		3.125	8.283582	3.089655	0.36632
M13	6.530769		41.8	0.604444	2.272727	4.169492	3.027027	95.25	5.550562				2.78135		103.3333			11.1194	1.510345	0.17064

D, day; D0, pre-vaccination; D7, seven days post-dose 1; D28, 28 days post-dose 1; D35, seven days post-dose 2; D56, 28 days post-dose 2; DENV, dengue virus; DPIV, tetravalent dengue purified inactivated vaccine; M, month; M7, six months post-dose 2; M13, 12 months post-dose 2; P, participant in each group with available data at any timepoint and DENV serotype.

Note: Changes are relative to D0 (pre-vaccination) values, after 6–7 days *in vitro* culture with combined DENV capsid/pre-membrane and envelope region peptide pools, following DPIV vaccination of dengue-naïve adults.

Supplementary Fig. 1 DPIV vaccination induces a multifunctional CD4+ T cell response.

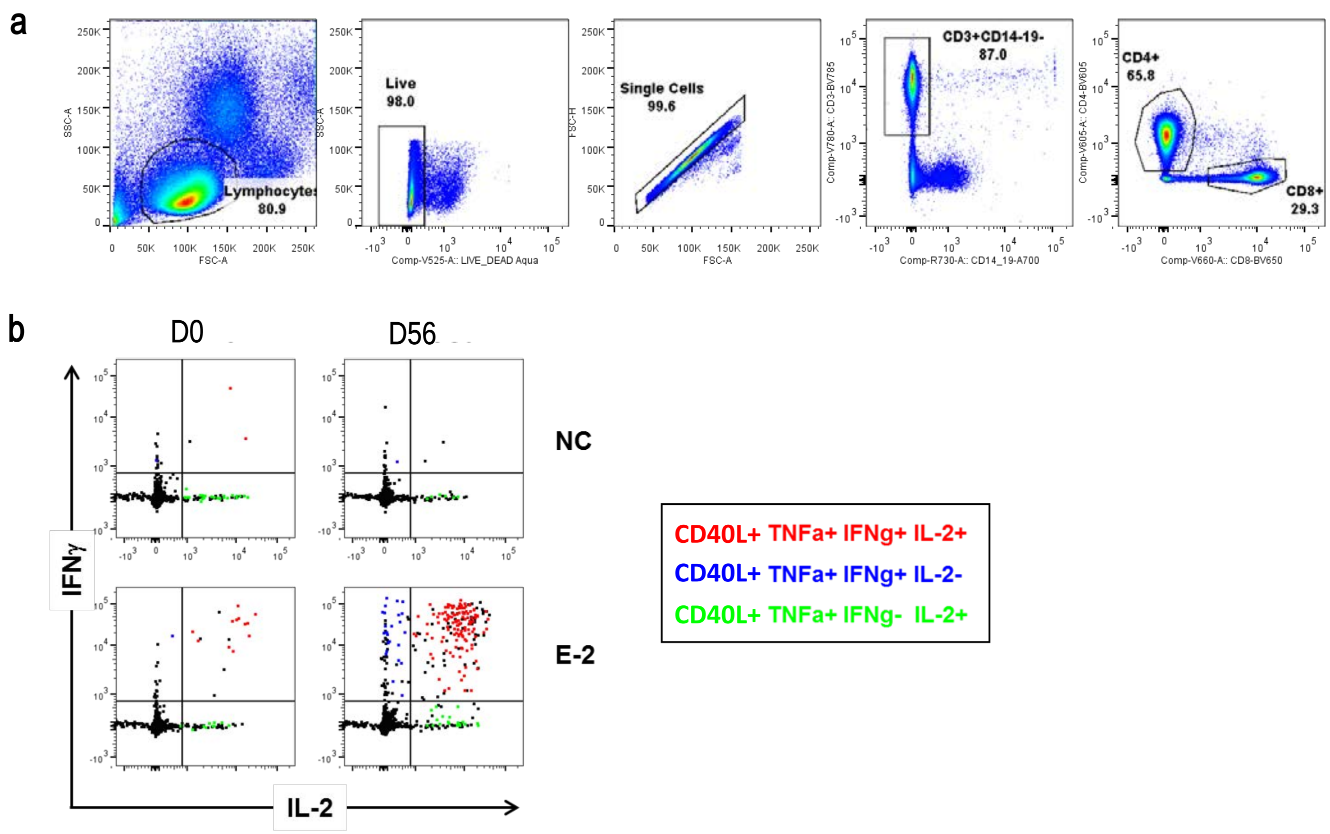

(a) Gating strategy to identify CD4+ and CD8+ T cell subsets. (b) Representative flow plots from a dengue-primed participant in study NCT01702857 showing IFN- versus IL-2 expression in CD4+ T cells after in vitro stimulation with peptide pools representing the E protein from DENV-2 (E-2). The participant whose data are represented in panel b had a pre-existing DENV-specific CD4+ T cell response, presumably from prior natural exposure that was substantially boosted by DPIV vaccination. CD, cluster differentiation; CD40L, cluster differentiation 40 ligand; day 0, pre-vaccination; day 56, 28 days post-dose 2; DENV, dengue virus; DPIV, tetravalent dengue purified inactivated vaccine; IFN, interferon; IL, interleukin; NC, negative control.

Supplementary Fig. 2. Multifunctional analysis of DPIV-specific CD4+ T cells

a

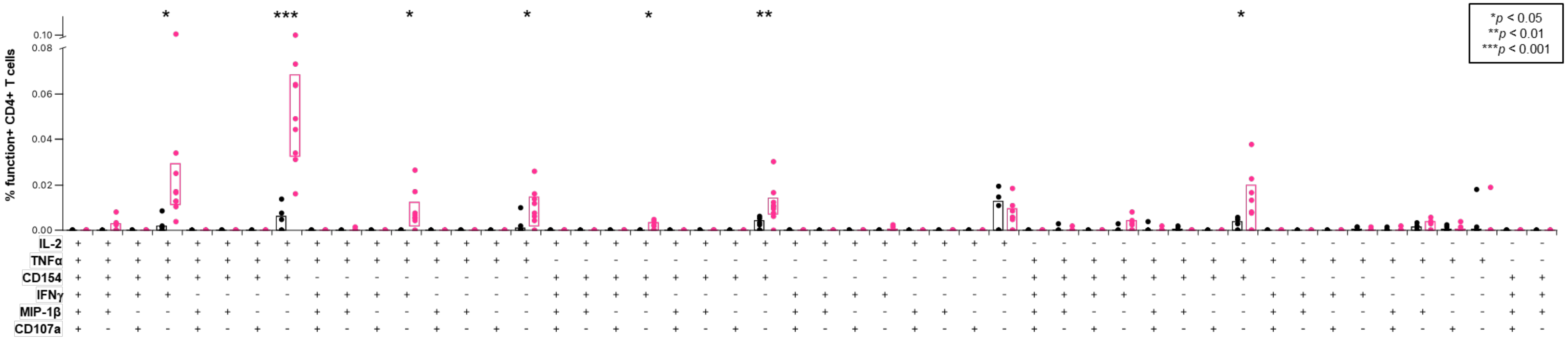

b

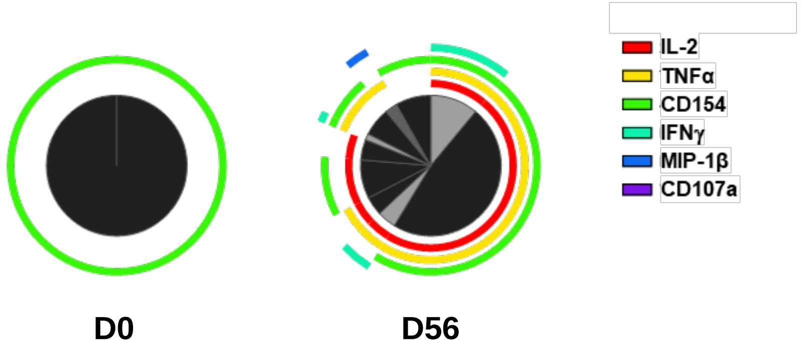

(a) The top responders to DPIV vaccination (N=9; selected for CME-specific responses to at least two DENV types that were ≥3 times the respective negative control) were selected to demonstrate the multifunctionality of DENV CME-specific CD4+ T cells elicited at day 56 post-vaccination (pink), as compared to pre-vaccination (black) responses. Boxes indicate interquartile range. Pre- versus post-vaccination responses were analyzed by Student's T test. (b) Pie charts show the average relative abundance of the different functional subpopulations. Data were background-subtracted, CM and E responses were combined, responses to DENV-1–4 were averaged, and then analyzed using SPICE v6.0 (Roederer, M., Nozzi, J. L. & Nason, M. C. SPICE: exploration and analysis of post-cytometric complex multivariate datasets. Cytometry A 79, 167-174 (2011)). CD, cluster differentiation; DPIV, tetravalent dengue purified inactivated vaccine; N, number of participants with data; CME, combined capsid/pre-membrane and envelope; DENV, dengue virus; IL, interleukin; TNF, tumor necrosis factor; IFN, interferon; MIP, macrophage inflammatory protein; D, day; D0, pre-vaccination; Day 56, 28 days post-dose 2.

Supplementary Fig. 3. Frequency of multifunctional CD4+ T cells expressing at least two functional markers, specific to dengue virus serotypes 1–4.

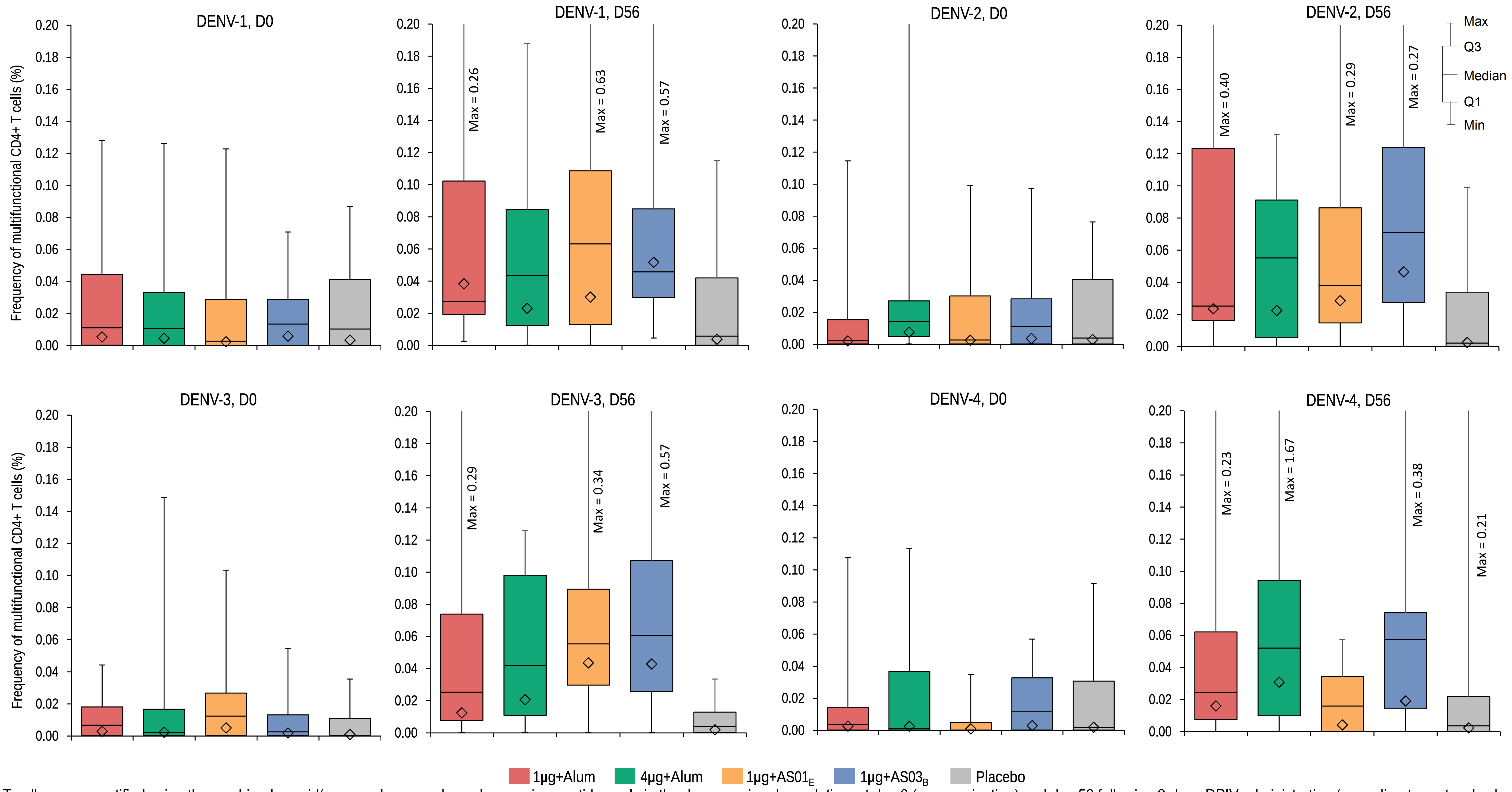

T cells were quantified using the combined capsid/pre-membrane and envelope region peptide pools in the dengue-primed population, at day 0 (pre-vaccination) and day 56 following 2-dose DPIV administration (according-to-protocol cohort for cell-mediated immunogenicity at day 56). Error bars represent Min and Max values in each group. CD, cluster differentiation; DPIV, tetravalent dengue purified inactivated vaccine; DENV, dengue virus; D, day; D0, pre-vaccination; D56, 28 days post-dose 2; DENV, dengue virus; DPIV, tetravalent dengue purified inactivated vaccine; Min/Max, minimum/maximum observation; Q1, Q3, first and third quartiles; ◊, geometric mean frequency. For each serotype, the combined capsid/ pre-membrane and envelope region peptide pools ((C+M)+E pool)-specific frequency was derived by adding the (C+M) pool specific frequency and the E pool specific frequency.

Supplementary Fig. 4 Frequencies of CD8+ T cells specific to dengue virus serotypes 1–4 expressing at least IL-2.

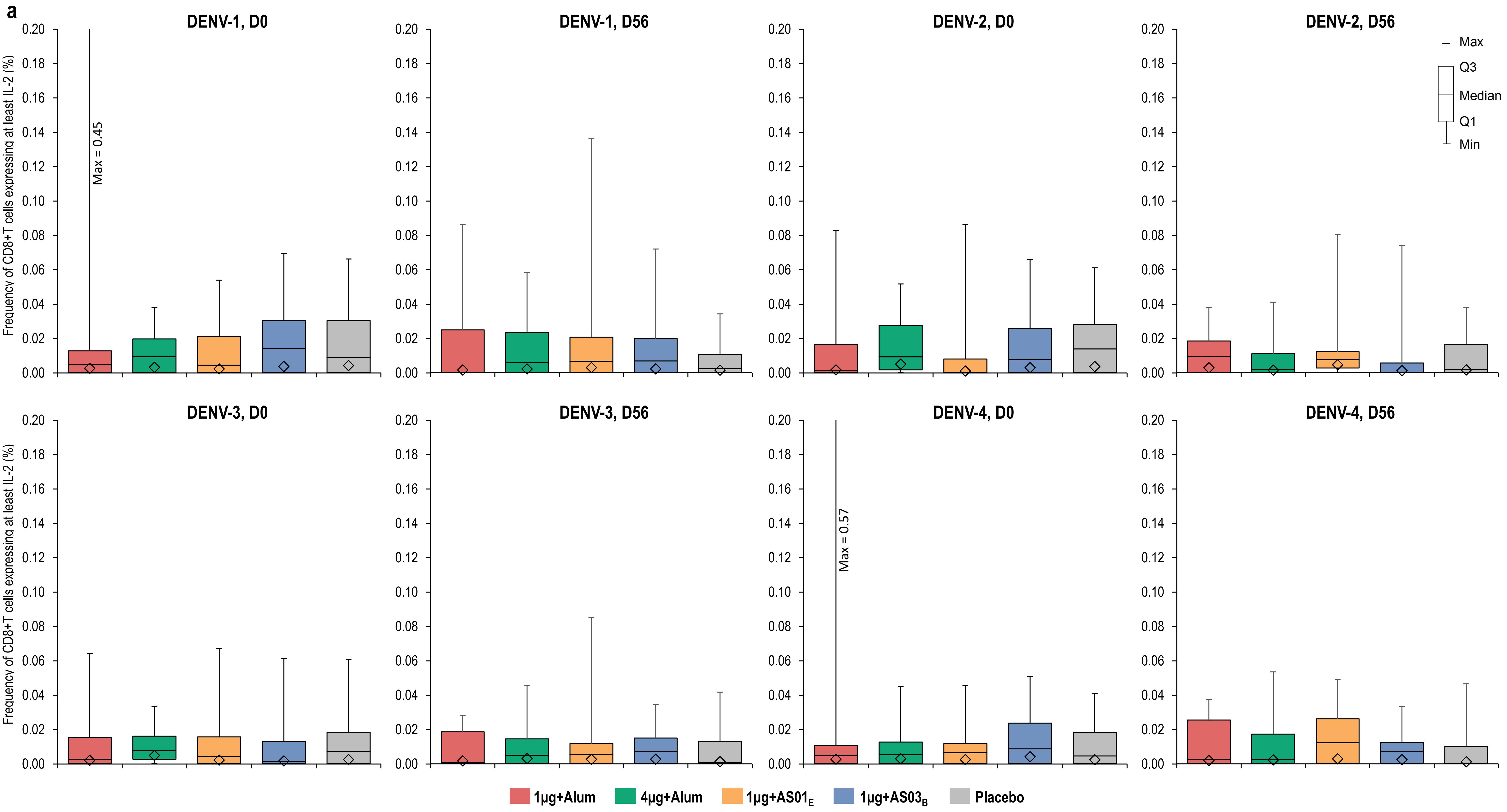

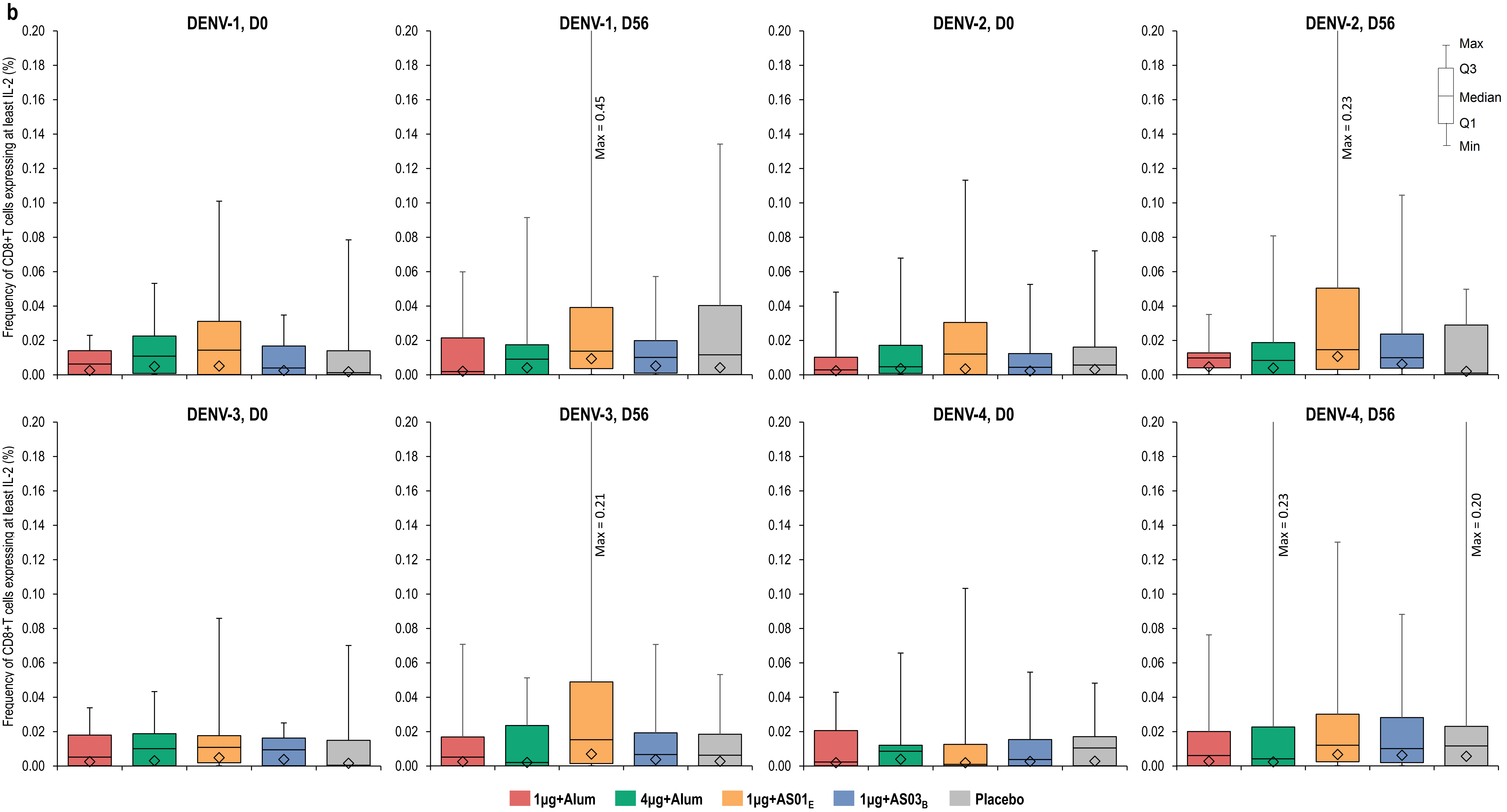

T cells were quantified using the combined capsid/pre-membrane and envelope region peptide pools in the predominantly dengue-naïve (a) and dengue-primed (b) populations (according-to-protocol cohort for cell-mediated immunogenicity at day 56), at day 0 (pre-vaccination) and day 56 following 2-dose DPIV administration. Individual values are plotted for each group. Error bars on the box p++lot represent the Min and Max values in each group. D, day; D0, pre-vaccination; D56, 28 days post-dose 2; DENV, dengue virus; DPIV, tetravalent dengue purified inactivated vaccine; Min/Max, minimum/maximum observation; Q1, Q3, first and third quartiles; ◊, geometric mean frequency. For each serotype, the combined capsid/pre-membrane and envelope region peptide pools ((C+M)+E pool)-specific frequency was derived by adding the (C+M) pool specific frequency and the E pool specific frequency.

Supplementary Fig. 5. Intracellular cytokine staining results for a subset of nine dengue-naïve adults, who received a DPIV booster dose.

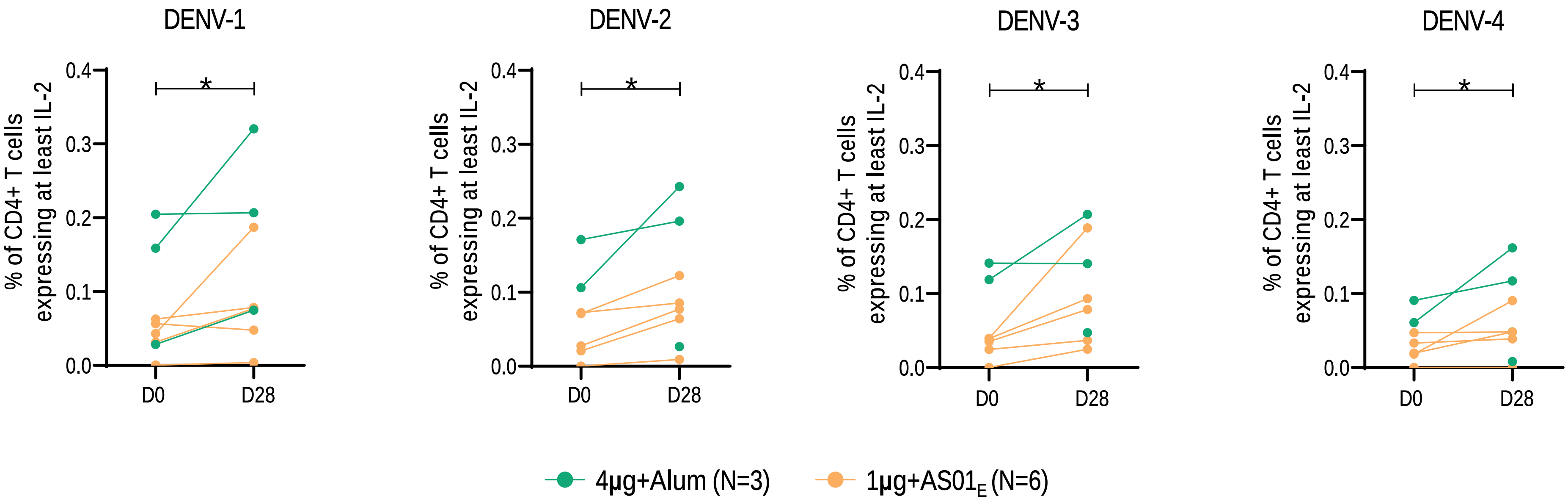

Each line represents the response of an individual participant pre-booster (D0) and 28 days (D28) post-booster. CD, cluster differentiation; DENV, dengue virus; DPIV, tetravalent dengue purified inactivated vaccine; IL, interleukin; N, number of participants who received a DPIV booster dose.
DPIV, tetravalent dengue purified inactivated vaccine; DENV, dengue virus; N, number of participants who received a DPIV booster dose. *p<0.05 by Wilcoxon.

Supplementary Fig. 6. T cell phenotyping analysis after 6–7-day in vitro culture using the combined capsid/pre-membrane and envelope region peptide pools.

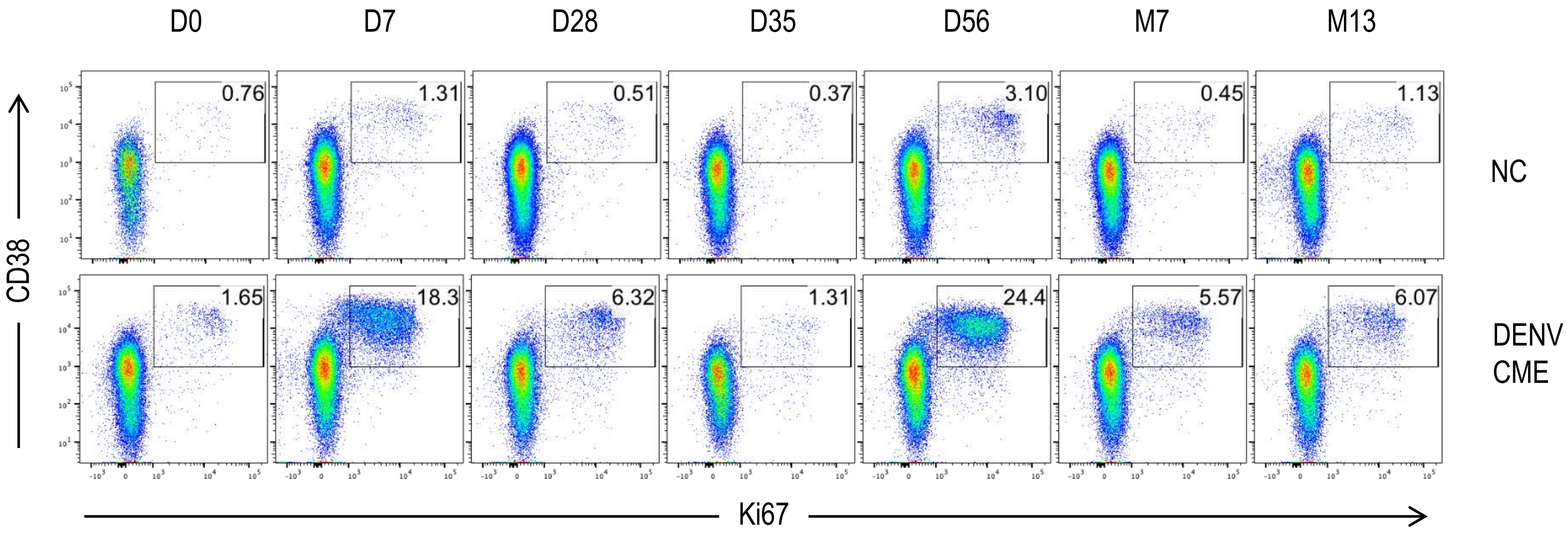

Representative flow plots show the frequency of activated (CD38+ Ki67+) CD4+ T cells of a single dengue-naïve study participant pre-vaccination (day 0) and at several time points post-vaccination. CD, cluster differentiation; CME, combined capsid/pre-membrane and envelope; D, day; D0, pre-vaccination; D7, seven days post-Dose 1; D28, 28 days post-Dose 1; D35, seven days post-dose 2; D56, 28 days post-Dose 2; DENV, dengue virus; M, month; M7, six months post-dose 2; M13, 12 months post-dose 2; NC, negative control.

Supplementary Fig. 7. Frequencies of memory B cells and neutralizing antibodies specific to dengue virus serotypes 1–4 in dengue-naïve individuals (adapted according-to-protocol cohort for immunogenicity).

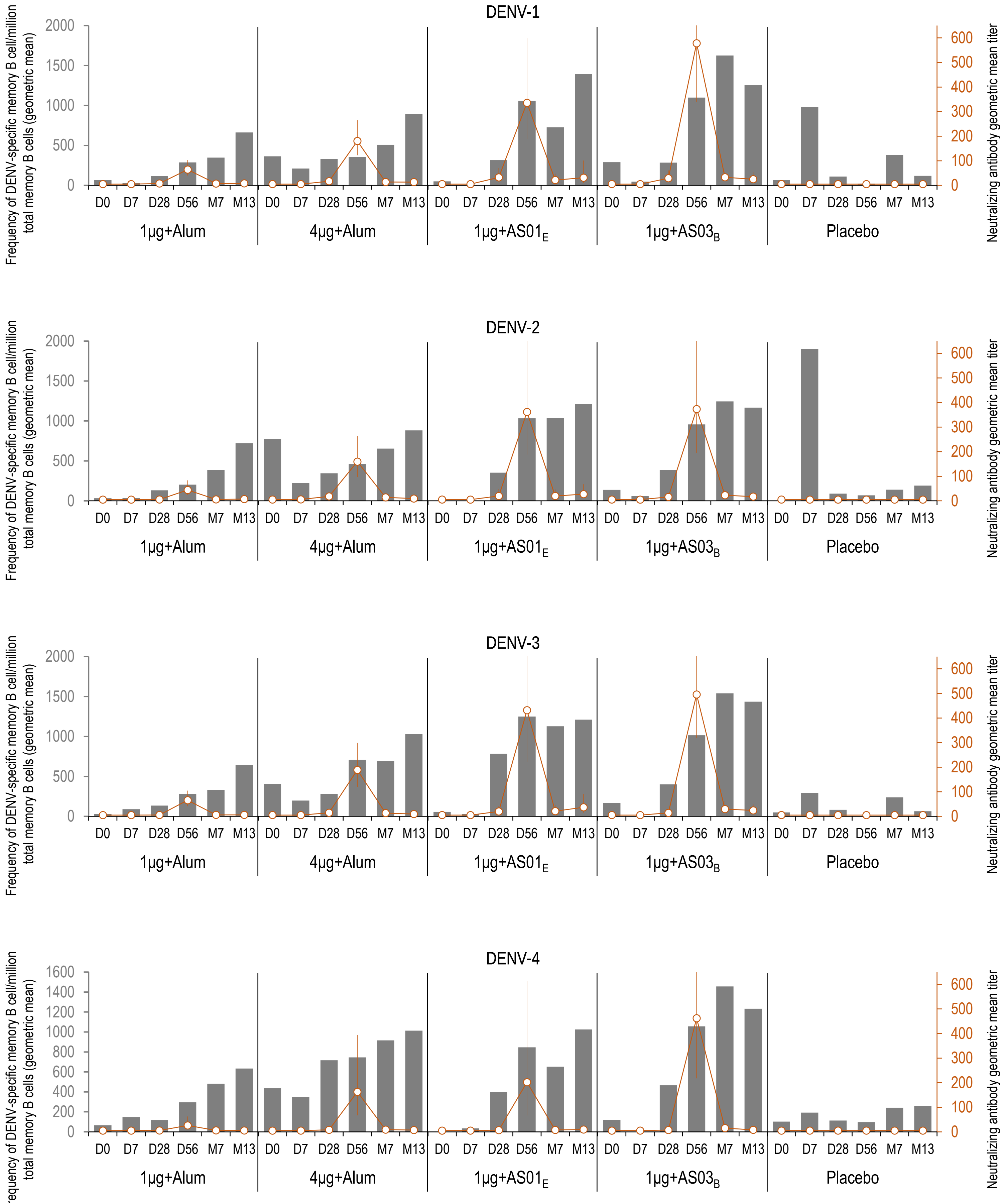

Bars represent the geometric mean of frequencies of memory B cells per million total memory B cells (primary vertical axis, left) and circles with error bars, the geometric mean titers for neutralizing antibodies to DENV 1–4 with 95% confidence intervals (secondary vertical axis, right). D, day; D0, pre-vaccination; D7, seven days post-dose 1; D28, 28 days post-dose 1; D35, seven days post-dose 2; D56, 28 days post-dose 2; DENV, dengue virus; DPIV, tetravalent dengue purified inactivated vaccine; M, month; M7, six months post-dose 2; M13, 12 months post-dose 2.
