# Supplementary material for: Characterization of B-cell and T-cell responses to a tetravalent dengue purified inactivated vaccine in healthy adults
Source: NPJ Vaccines. 2022 Oct 31;7:132. doi: 10.1038/s41541-022-00537-2 (PMC9622737; doi:10.1038/s41541-022-00537-2)
Supplement: Supplementary file 2 — REPORTING SUMMARY [file 41541_2022_537_MOESM2_ESM.pdf]

nature portfolio

Corresponding author(s): Heather Friberg

Last updated by author(s): Sep 2, 2022

Reporting Summary

Nature Portfolio wishes to improve the reproducibility of the work that we publish. This form provides structure for consistency and transparency in reporting. For further information on Nature Portfolio policies, see our Editorial Policies and the Editorial Policy Checklist.

Statistics

For all statistical analyses, confirm that the following items are present in the figure legend, table legend, main text, or Methods section.

n/a	Confirmed	
☐	☒	The exact sample size (*n*) for each experimental group/condition, given as a discrete number and unit of measurement
☐	☒	A statement on whether measurements were taken from distinct samples or whether the same sample was measured repeatedly
☒	☐	The statistical test(s) used AND whether they are one- or two-sided<br>*Only common tests should be described solely by name; describe more complex techniques in the Methods section.*
☒	☐	A description of all covariates tested
☒	☐	A description of any assumptions or corrections, such as tests of normality and adjustment for multiple comparisons
☐	☒	A full description of the statistical parameters including central tendency (e.g. means) or other basic estimates (e.g. regression coefficient) AND variation (e.g. standard deviation) or associated estimates of uncertainty (e.g. confidence intervals)
☒	☐	For null hypothesis testing, the test statistic (e.g. *F*, *t*, *r*) with confidence intervals, effect sizes, degrees of freedom and *P* value noted<br>*Give P values as exact values whenever suitable.*
☒	☐	For Bayesian analysis, information on the choice of priors and Markov chain Monte Carlo settings
☒	☐	For hierarchical and complex designs, identification of the appropriate level for tests and full reporting of outcomes
☒	☐	Estimates of effect sizes (e.g. Cohen's *d*, Pearson's *r*), indicating how they were calculated

Our web collection on statistics for biologists contains articles on many of the points above.

Software and code

Policy information about availability of computer code

Data collection	BD LSRFortessa flow cytometer (BD Biosciences); MAGPIX (Bio-Rad)
Data analysis	FlowJo (Becton, Dickinson & Company); Microsoft Excel (Microsoft Corp.); and GraphPad Prism (GraphPad Software).

For manuscripts utilizing custom algorithms or software that are central to the research but not yet described in published literature, software must be made available to editors and reviewers. We strongly encourage code deposition in a community repository (e.g. GitHub). See the Nature Portfolio guidelines for submitting code & software for further information.

Data

Policy information about availability of data

All manuscripts must include a data availability statement. This statement should provide the following information, where applicable:

- Accession codes, unique identifiers, or web links for publicly available datasets
- A description of any restrictions on data availability
- For clinical datasets or third party data, please ensure that the statement adheres to our policy

Information on GSK's data sharing commitments and requesting access to anonymized individual participant data and associated documents from GSK sponsored studies can be found at www.clinicalstudydatarequest.com (study ID: 116289/116614). To access data for other types of GSK sponsored studies, visit www.clinicalstudydatarequest.com. Questions regarding information on WRAIR data sharing commitments and access can be directed to the WRAIR Research Programs Office.

Field-specific reporting

Please select the one below that is the best fit for your research. If you are not sure, read the appropriate sections before making your selection.

☒ Life sciences ☐ Behavioural & social sciences ☐ Ecological, evolutionary & environmental sciences

For a reference copy of the document with all sections, see nature.com/documents/nr-reporting-summary-flat.pdf

Life sciences study design

All studies must disclose on these points even when the disclosure is negative.

Sample size	Descriptive studies. A sample size of 20 participants per group was chosen. This corresponds to a 95% CI of 0%-16.8% for the detection of AEs in at least 5% of participants in each group. No sample size calculations in relation to a specific objective or for the Dose 3 Subset were performed in study NCT01702857.
Data exclusions	Analyses were performed in all evaluable participants who had data available for the evaluated immunogenicity endpoint at each time point. To add: selection of participants for activated CD38+ Ki67+ CD4+ T cells among total CD4+ T cells?
Replication	The reproducibility of data was built into the experimental design through testing of multiple subjects per vaccine group, and comparing the group means.
Randomization	participants were randomized 1:1:1:1:1 to receive two doses of different formulations of DPIV, administered four weeks apart (on days 0 and 28): 1 µg/serotype/dose adjuvanted with either alum (Group 1 µg + Alum), AS01E (Group 1 µg + AS01E), or AS03B (Group 1 µg + AS03B), 4 µg/serotype/dose adjuvanted with alum (Group 4 µg + Alum), or placebo.
Blinding	Observer blind trial, with the vaccine/placebo recipients and those responsible for the evaluation of all study endpoints being unaware of treatment assignments. Vaccine preparation was performed at the WRAIR Pilot Bio-production Facility and administration of the vaccine was done at the WRAIR CTC by nurse coordinators who did not participate in any of the clinical study evaluation activities.<br>The laboratories in charge of testing were blinded with regard to treatment allocation, and codes were used to link the subject and study to each sample.

Reporting for specific materials, systems and methods

We require information from authors about some types of materials, experimental systems and methods used in many studies. Here, indicate whether each material, system or method listed is relevant to your study. If you are not sure if a list item applies to your research, read the appropriate section before selecting a response.

Materials & experimental systems

n/a	Involved in the study
☐	☒ Antibodies
☐	☐ Eukaryotic cell lines
☐	☐ Palaeontology and archaeology
☐	☐ Animals and other organisms
☐	☒ Human research participants
☐	☐ Clinical data
☐	☐ Dual use research of concern

Methods

n/a	Involved in the study
☐	☐ ChIP-seq
☐	☒ Flow cytometry
☐	☐ MRI-based neuroimaging

Antibodies

Antibodies used	ELISPOT assay: AffiniPure Goat anti human IgG (H+L) (Jackson Laboratories 109.005.003; Polyclonal AB_2337532; lot #108845, 111755, 111755, 113660); Biotin SP conjugated AffiniPure Goat anti-human IgG (Jackson Laboratories 109.065.008; Polyclonal AB_2337623; lot #92812)<br>ICS Assay: CD3-BV785 (BioLegend 317329; clone: OKT3; lot #B155661, B169980, B177199, B188394); CD4-BV605 (BioLegend 317437/317438; clone: OKT4; lot #B158711, B176892, B185762, B189706); CD8a-BV650 (BioLegend 301041; clone: RPA-T8; lot #B150802, B155883, B172154, B180800, B186467); CD14-AlexaFluor700 (BD Pharmingen 557923; clone: M5E2; lot #39470, 4045984, 2346722, 4273566); CD19-AlexaFluor700 (BD Pharmingen 557921; clone: HIB19; lot #34288, 3213842, 4163728); IFN-gamma-eFluor450 (eBioscience 48-7319-42; clone: 4S.B3, lot #E10948-1632, E10948-1633, E10948-1635); TNF-alpha-PE-Cy7 (eBioscience 25-7349-82; clone: MAb11, lot #E07679-1630, E07679-1631, E07679-1633); MIP-1beta-PE (BD Pharmingen 550078; clone: D21-1351, lot #15874, 3224656, 4189575); IL-2-APC (BD Pharmingen 554567; clone: MQ1-17H12; lot #13860, 3207970, 4247735); CD107a-FITC (BD Pharmingen 555800; clone: H4A3; lot #23139, 4042957); CD154-PE-Cy5 (BD Pharmingen 555701; clone: TRAP1; lot #23740, 3179996, 4169819); CD28 (BD Pharmingen 555725; clone: CD28.2; lot #30558, 3161568, 3011734); CD49d (BD Pharmingen 555501; clone: 9F10; lot #99120, 3018506)<br>7-day stimulation panel: CD3-BV785 (BioLegend 317329; clone: OKT3; lot #B155661, B169980, B177199, B188394); CD4-BV605 (BioLegend 317437/317438; clone: OKT4; lot #B158711, B176892, B185762, B189706); CD8--AlexaFluor700 (BD Pharmingen 557945;

clone: RPA-T8); CD14-BV510 (BD Horizon 563079; clone: MΦP9); CD19-BV510 (BD Horizon 562947; clone: SJ25C1); CD56-PE-Cy7 (BD Pharmingen 557747, clone: B159); CD16-APC-Cy7 (BioLegend 302018, clone: 3G8); CD38-PE (Biolegend 356604, clone: HB-7); HLA-DR-PE-Dazzle594 (BioLegend 307654, clone: L243); Ki67-AlexaFluor488 (BD Pharmingen 561165, clone: B56).

Validation

All antibodies used were commercially purchased reagents validated and sold for research use only.

Eukaryotic cell lines

Policy information about cell lines

Cell line source(s)

State the source of each cell line used.

Authentication

Describe the authentication procedures for each cell line used OR declare that none of the cell lines used were authenticated.

Mycoplasma contamination

Confirm that all cell lines tested negative for mycoplasma contamination OR describe the results of the testing for mycoplasma contamination OR declare that the cell lines were not tested for mycoplasma contamination.

Commonly misidentified lines
(See ICLAC register)

Name any commonly misidentified cell lines used in the study and provide a rationale for their use.

Palaeontology and Archaeology

Specimen provenance

Provide provenance information for specimens and describe permits that were obtained for the work (including the name of the issuing authority, the date of issue, and any identifying information). Permits should encompass collection and, where applicable, export.

Specimen deposition

Indicate where the specimens have been deposited to permit free access by other researchers.

Dating methods

If new dates are provided, describe how they were obtained (e.g. collection, storage, sample pretreatment and measurement), where they were obtained (i.e. lab name), the calibration program and the protocol for quality assurance OR state that no new dates are provided.

☐ Tick this box to confirm that the raw and calibrated dates are available in the paper or in Supplementary Information.

Ethics oversight

Identify the organization(s) that approved or provided guidance on the study protocol, OR state that no ethical approval or guidance was required and explain why not.

Note that full information on the approval of the study protocol must also be provided in the manuscript.

Animals and other organisms

Policy information about studies involving animals; ARRIVE guidelines recommended for reporting animal research

Laboratory animals

For laboratory animals, report species, strain, sex and age OR state that the study did not involve laboratory animals.

Wild animals

Provide details on animals observed in or captured in the field; report species, sex and age where possible. Describe how animals were caught and transported and what happened to captive animals after the study (if killed, explain why and describe method; if released, say where and when) OR state that the study did not involve wild animals.

Field-collected samples

For laboratory work with field-collected samples, describe all relevant parameters such as housing, maintenance, temperature, photoperiod and end-of-experiment protocol OR state that the study did not involve samples collected from the field.

Ethics oversight

Identify the organization(s) that approved or provided guidance on the study protocol, OR state that no ethical approval or guidance was required and explain why not.

Note that full information on the approval of the study protocol must also be provided in the manuscript.

Human research participants

Policy information about studies involving human research participants

Population characteristics

NCT01666652: The mean age in the total vaccinated cohort at first vaccination was 27.8 years (median = 27 years; range = 18–39 years); 43% of participants were female; 51% self-identified as African American; 34% as Caucasian. The mean age in the booster total vaccinated cohort at first vaccination was 27.8 years (median = 26 years; range = 21–37 years); 33% of participants were female; 67% were African American.
NCT01702857: The mean age in the total vaccinated cohort at first vaccination was 27.9 years; 61% of participants were female. All participants were American Hispanic or Latino.
and 33% Caucasian.

Recruitment

NCT01666652: Healthy male and female adults between 18 and 39 years of age were recruited by staff from the WRAIR CTC. Volunteers were provided with a detailed explanation of the study and enrolled after an informed consent process. Female participants had to be of nonchildbearing potential or abstinent, or had to use adequate contraceptive precautions for 30

	days prior to vaccination, had a negative pregnancy test on the day of vaccination, and agreed to continue such precautions for 60 days after completion of the vaccination series. Volunteers initially seropositive for hepatitis B surface antigen, hepatitis C virus antibodies, or human immunodeficiency virus antibodies were excluded. Other exclusion criteria were a history of chronic disease, chronic alcohol consumption and/or drug abuse, receipt of immunoglobulins and/or any blood products within 90 days preceding vaccination or planned administration during the study period, as well as clinically significant laboratory abnormalities. Additionally, participants were excluded from the booster phase if they had received any vaccination within 4 weeks prior to booster vaccination.<br>NCT01702857: Healthy male and female adults between 18 and 39 years of age who have lived in the Caribbean for more than 10 years were recruited at the University of Puerto Rico Medical Sciences Campus, Puerto Rico Clinical and Translational Research Consortium Center. Volunteers were provided with a detailed explanation of the study and enrolled after an informed consent process. Female participants had to be of nonchildbearing potential or abstinent, or had to use adequate contraceptive precautions for 30 days before vaccination, have a negative pregnancy test on the day of vaccination, and agreed to continue such precautions for 60 days after completion of the vaccination series. Volunteers seropositive for hepatitisBsurface antigen, hepatitis C virus antibodies, or human immunodeficiency virus antibodieswere excluded.Other exclusion criteriawere a history of chronic disease; chronic alcohol consumption and/or drug abuse; and receipt of immunoglobulins and/or any blood products within 90 days preceding vaccination or had planned administration during the study period and laboratory test results outside normal limits for age, gender, and locality, at screening.
Ethics oversight	The study protocols, protocol amendments, informed consents, and other information that required pre-approval were reviewed and approved by the Walter Reed Army Institute of Research (WRAIR) Institutional Review Board.

Note that full information on the approval of the study protocol must also be provided in the manuscript.

Clinical data

Policy information about clinical studies

All manuscripts should comply with the ICMJE guidelines for publication of clinical research and a completed CONSORT checklist must be included with all submissions.

Clinical trial registration	NCT01666652; NCT01702857
Study protocol	For reasons of privacy protection for study participants, GSK offers access to data and materials via controlled access. Anonymized individual participant data from this study plus the annotated case report form, protocol, reporting and analysis plan, dataset specifications, raw dataset, analysis-ready dataset, and clinical study report are available for research proposals approved by an independent review committee. Proposals should be submitted to www.clinicalstudydatarequest.com (study identifier: 116289/116614). A data access agreement will be required.
Data collection	NCT01666652: United States, Maryland; WRAIR, Clinical Trials Center; study conducted between Sept 2012 and Nov 2017<br>NCT01702857: Puerto Rico, Clinical Research Center, 1st Floor University Hospital; study conducted between Nov 2012 and March 2017
Outcomes	Exploratory outcomes ar described here

Dual use research of concern

Policy information about dual use research of concern

Hazards

Could the accidental, deliberate or reckless misuse of agents or technologies generated in the work, or the application of information presented in the manuscript, pose a threat to:

No	Yes	
☒	☐	Public health
☒	☐	National security
☒	☐	Crops and/or livestock
☒	☐	Ecosystems
☒	☐	Any other significant area

Experiments of concern

Does the work involve any of these experiments of concern:

No	Yes	
☒	☐	Demonstrate how to render a vaccine ineffective
☒	☐	Confer resistance to therapeutically useful antibiotics or antiviral agents
☒	☐	Enhance the virulence of a pathogen or render a nonpathogen virulent
☒	☐	Increase transmissibility of a pathogen
☒	☐	Alter the host range of a pathogen
☒	☐	Enable evasion of diagnostic/detection modalities
☒	☐	Enable the weaponization of a biological agent or toxin
☒	☐	Any other potentially harmful combination of experiments and agents

ChIP-seq

Data deposition

☐ Confirm that both raw and final processed data have been deposited in a public database such as GEO.

☐ Confirm that you have deposited or provided access to graph files (e.g. BED files) for the called peaks.

Data access links<br>*May remain private before publication.*	*For "Initial submission" or "Revised version" documents, provide reviewer access links. For your "Final submission" document, provide a link to the deposited data.*
Files in database submission	*Provide a list of all files available in the database submission.*
Genome browser session<br>(e.g. UCSC)	*Provide a link to an anonymized genome browser session for "Initial submission" and "Revised version" documents only, to enable peer review. Write "no longer applicable" for "Final submission" documents.*

Methodology

Replicates	*Describe the experimental replicates, specifying number, type and replicate agreement.*
Sequencing depth	*Describe the sequencing depth for each experiment, providing the total number of reads, uniquely mapped reads, length of reads and whether they were paired- or single-end.*
Antibodies	*Describe the antibodies used for the ChIP-seq experiments; as applicable, provide supplier name, catalog number, clone name, and lot number.*
Peak calling parameters	*Specify the command line program and parameters used for read mapping and peak calling, including the ChIP, control and index files used.*
Data quality	*Describe the methods used to ensure data quality in full detail, including how many peaks are at FDR 5% and above 5-fold enrichment.*
Software	*Describe the software used to collect and analyze the ChIP-seq data. For custom code that has been deposited into a community repository, provide accession details.*

Flow Cytometry

Plots

Confirm that:

☒ The axis labels state the marker and fluorochrome used (e.g. CD4-FITC).

☒ The axis scales are clearly visible. Include numbers along axes only for bottom left plot of group (a 'group' is an analysis of identical markers).

☒ All plots are contour plots with outliers or pseudocolor plots.

☒ A numerical value for number of cells or percentage (with statistics) is provided.

Methodology

Sample preparation	PBMC were isolated from whole blood specimens and cryopreserved for batched testing. Refer to the Methods section for additional details.
Instrument	LSRFortessa (BD Biosciences)

Software	Data collected using FACS Diva (v7) and analyzed using FlowJo (v7)
Cell population abundance	No sorting was done for these experiments
Gating strategy	As shown in Supplementary Figure 1, antigen-specific cells were first identified by forward and side scatter profiles, then selected for the absence of staining with a viability dye. Then forward scatter area versus height was used to select single cells, followed by gating on CD3+ cells which did not also express CD14 or CD19. Subsequently, CD4+ and CD8+ T cell sub-populations were identified and evaluated for the presence of the 6 different functional markers (IL-2, IFN-gamma, TNF-alpha, CD154, CD107a, MIP-1beta).

☒ Tick this box to confirm that a figure exemplifying the gating strategy is provided in the Supplementary Information.

Magnetic resonance imaging

Experimental design

Design type	*Indicate task or resting state; event-related or block design.*
Design specifications	*Specify the number of blocks, trials or experimental units per session and/or subject, and specify the length of each trial or block (if trials are blocked) and interval between trials.*
Behavioral performance measures	*State number and/or type of variables recorded (e.g. correct button press, response time) and what statistics were used to establish that the subjects were performing the task as expected (e.g. mean, range, and/or standard deviation across subjects).*

Acquisition

Imaging type(s)	*Specify: functional, structural, diffusion, perfusion.*
Field strength	*Specify in Tesla*
Sequence & imaging parameters	*Specify the pulse sequence type (gradient echo, spin echo, etc.), imaging type (EPI, spiral, etc.), field of view, matrix size, slice thickness, orientation and TE/TR/flip angle.*
Area of acquisition	*State whether a whole brain scan was used OR define the area of acquisition, describing how the region was determined.*

Diffusion MRI ☐ Used ☐ Not used

Preprocessing

Preprocessing software	*Provide detail on software version and revision number and on specific parameters (model/functions, brain extraction, segmentation, smoothing kernel size, etc.).*
Normalization	*If data were normalized/standardized, describe the approach(es): specify linear or non-linear and define image types used for transformation OR indicate that data were not normalized and explain rationale for lack of normalization.*
Normalization template	*Describe the template used for normalization/transformation, specifying subject space or group standardized space (e.g. original Talairach, MNI305, ICBM152) OR indicate that the data were not normalized.*
Noise and artifact removal	*Describe your procedure(s) for artifact and structured noise removal, specifying motion parameters, tissue signals and physiological signals (heart rate, respiration).*
Volume censoring	*Define your software and/or method and criteria for volume censoring, and state the extent of such censoring.*

Statistical modeling & inference

Model type and settings	*Specify type (mass univariate, multivariate, RSA, predictive, etc.) and describe essential details of the model at the first and second levels (e.g. fixed, random or mixed effects; drift or auto-correlation).*
Effect(s) tested	*Define precise effect in terms of the task or stimulus conditions instead of psychological concepts and indicate whether ANOVA or factorial designs were used.*

Specify type of analysis: ☐ Whole brain ☐ ROI-based ☐ Both

Statistic type for inference (See Eklund et al. 2016)	*Specify voxel-wise or cluster-wise and report all relevant parameters for cluster-wise methods.*
Correction	*Describe the type of correction and how it is obtained for multiple comparisons (e.g. FWE, FDR, permutation or Monte Carlo).*

Models & analysis

n/a	Involved in the study
☒	☐ Functional and/or effective connectivity
☒	☐ Graph analysis
☒	☐ Multivariate modeling or predictive analysis
